# Studies of *Piper auritum* Kuntz’s Mutagenic and Antimutagenic Properties Using the Ames Test

**DOI:** 10.3390/metabo15030164

**Published:** 2025-03-01

**Authors:** Luis S. Muñoz-Carrillo, Eduardo Madrigal-Bujaidar, Sandra L. Hernández-Ojeda, José A. Morales-González, Eduardo O. Madrigal-Santillán, Isela Álvarez-González, J. Javier Espinosa-Aguirre

**Affiliations:** 1 Laboratorio de Genética, Escuela Nacional de Ciencias Biológicas, Instituto Politécnico Nacional, Unidad Profesional Adolfo López Mateos, Zacatenco, Av. Wilfrido Massieu s/n, Gustavo A. Madero, Ciudad de Mexico 07738, Mexico; louismcar90@gmail.com (L.S.M.-C.); edumadrigal.bujaidar@gmail.com (E.M.-B.); 2 Instituto de Investigaciones Biomédicas, Universidad Nacional Autónoma de México, Tercer Circuito Exterior s/n, Ciudad Universitaria, Ciudad de Mexico 04510, Mexico; slhernandez@iibiomedicas.unam.mx; 3 Laboratorio de Medicina de la Conservación, Escuela Superior de Medicina, Instituto Politécnico Nacional, Plan de San Luis y Díaz Mirón s/n, Casco de Santo Tomás, Ciudad de Mexico 11340, Mexico; jmorales101@yahoo.com.mx (J.A.M.-G.); emadrigal@ipn.mx (E.O.M.-S.)

**Keywords:** Ames test, CYP1A1, docking studies, mutagenicity/antimutagenicity, *Piper auritum*, safrole

## Abstract

Background: *Piper auritum* Kuntz is an endemic plant from Mexico and Central America, where it is called “hoja santa”, and it is widely used in both local cuisine and traditional medicine. By using the Ames test (strain TA98), we recently demonstrated that ethanol extract from the plant has no mutagenic potential and that it has a significant antimutagenic effect. Objectives/Methods: In the present report, we extended this evaluation by using five strains of the *Salmonella*/microsome mutagenicity assay. Moreover, we evaluated the mutagenic/antimutagenic potential of the major component of the ethanol extract, safrole, with the TA98 strain and employed docking studies to examine the molecular relationship of safrole with the CYP1A1 isoform. Results: Our results confirmed the absence of mutagenicity in the ethanol plant extract, as well as a concentration-dependent inhibitory effect on the damage induced by benzo[a]pyrene (BaP). With respect to safrole, we also determined that the compound has no mutagenic potential and has a strong inhibitory effect on the damage induced by BaP. Docking and kinetic analysis confirmed the coupling of safrole with the active site of the CYP1A1 enzyme, leading to competitive inhibition. Conclusions: These results suggest that the inhibitory effect on the enzyme activity is one of the possible antimutagenic mechanisms.

## 1. Introduction

Plants have been used for human welfare since ancient times, particularly in areas related to food and health. In modern life, the use of plants in traditional medicine has increased worldwide, which is supported mainly by increased knowledge of the health benefits produced by vegetal secondary metabolites, as well as by the development of plant-derived molecules with effects related to microbiological or immunological problems, diabetes, cancer, or pain, among other health problems [1]. In this field, investigations of different types of plant extracts and their constituents have yielded useful results in toxicological and pharmacological areas, providing information about safe or unsafe doses; the duration of exposure; and the effects induced at the molecular, cellular and clinical levels. The *Piper* genus comprises more than 2000 species, with about 40 of them being more frequently mentioned in the literature. Among these, it has been reported that *P. nigrum*, *P. longum*, and *P. betle* are the most cited, along with another 27 species, including *Piper auritum* [2,3,4]. Some authors have addressed their use as condiments in a number of dishes as well as the presence of carbohydrates, proteins, minerals, vitamins, fats and essential oils in these plants. Their use in folk medicine to treat fever, diarrhea, rheumatism, gastrointestinal distress, cancer, pain and inflammation, as well as their properties as anthelmintics, diuretics, and for controlling high blood pressure, has also been described. All these biological activities are related to the presence of bioactive compounds such as amides, alkaloids, flavonoids, tannins, saponins, glycosides, terpenoids, and phenolic compounds.

The present report refers to *Piper auritum*, a plant known as “sacred leaf” or “hoja santa” in Mexico. The studied plant, which belongs to the Piperaceae family, is found from southern Mexico to Colombia and presently grows in tropical regions of the world; it is a bush from 1.5 to 6 m in height, with erect stems and ovate, oblong or cordiform leaves. Their leaves are used in the preparation of different dishes in Mexican cuisine, as well as in traditional medicine, because of their properties as diuretics and antipyretics; for the treatment of gout, angina pectoris and venereal diseases; and because of their properties as local anesthetic and appetite stimulant [5].

Pharmacological in vitro studies of the plant extract have shown to have antibacterial, cytotoxic and antioxidant properties, particularly in the last case, as indicated by DPPH and ABTS assays. Additionally, in vivo studies involving administration of a hexanic extract of *Piper auritum* revealed a significant reduction in the levels of glucose, cholesterol and triglycerides; moreover, other reports have demonstrated an anti-inflammatory effect of the extract because of a significant reduction in carrageenan-induced edema [6,7,8].

The main component of the plant is safrole, 5-(2-propenyl)-1,3-benzodioxone, a chemical that is also present in other plants, such as sassafras, camphor, nutmeg, and black pepper; it is used in the food industry as flavoring and in dentistry as a component of toothpastes [9]. Studies on this agent have revealed that it can cause liver rodent toxicity due to CYP-induced electrophile metabolites that may attack DNA and can be involved in tumor induction; however, in contrast to this deleterious effect, safrole has also been reported to be an antioxidant (as shown by DPPH assay results), an antidiabetic agent as shown by measuring amilase inhibitory effects, an antimicrobial agent that is effective against a number of bacteria, and a cytotoxic agent against Hep3B hepatocellular carcinoma cells, which suggests anticarcinogenic potential [10]. Moreover, mutagenic studies with the Ames test and the mouse bone marrow micronuclei assay have yielded negative results [11].

When considering the safety of food- and health-related products, evaluating their genotoxic potential is important because of the well-known relationship between DNA damage and molecular and cellular alterations that may cause clinical anomalies. In this context, a commonly used test to examine genotoxic potential is the *Salmonella typhimurium*/microsome assay, which is a highly useful short-term test for determining the generation of point mutations [12,13].

*Piper auritum* is known to be widely utilized in various countries both as food and as a curative agent in traditional medicine. The plant possesses nutritional value and pharmacological effects; therefore, it merits further research about its properties, especially because the plant contains safrole, a probable carcinogen. Interestingly, by using the strain TA98 of *S. typhimurium*, our laboratory demonstrated no mutagenicity of an ethanol plant extract and safrole. However, a strong antimutagenic potential against heterocyclic aromatic amines was found for both [14]. In order to validate these data, we have confirmed the absence of mutagenicity of the plant extract by using five strains of *S. typhimurium*, as internationally recommended [15], and determined a strong antimutagenic effect of safrole against BaP using the TA98 strain of *S. typhimurium.* Considering that BaP is a premutagenic aromatic hydrocarbon requiring CYP1A1 biotransformation to be activated, we were able to examine CYP1A1 inhibition as a possible mechanism of antimutagenicity for the plant extract and safrole.

## 2. Materials and Methods

### 2.1. Chemicals

Benzo[a]pyrene (BaP), 2-nitrofluorene (2NF), 4-nitroquinoline N-oxide (4-NQO), NADPH, γ-terpinene, caryophyllene, pentadecane, sodium azide, 2-aminoanthracene (2-AA), β-naphthoflavone, 7-ethoxyresorufin, 7-methoxyresorufin, resorufin, and picrolonic acid were obtained from Sigma–Aldrich (St. Louis, MO, USA). Dimethylsulfoxide (DMSO) was purchased from Merck (Darmstadt, Germany), α-copaene was obtained from Cayman Chemical Company (Ann Arbor, MI, USA), phenobarbital was obtained from Abbot Laboratories de México, S.A. de C.V., and safrole was obtained from US Biological Life Sciences (Swampscott, MA, USA). Nutrient broth N° 2 was obtained from OXOID (Basingstoke, UK). Bacto Agar was obtained from BD (Le Pont de Claix, France).

### 2.2. Plant Collection and Extract Preparation

The plant sample was collected in June 2023 from a free-pesticide cultivar in southern Mexico City, and a voucher specimen was deposited at the herbarium of the School of Sciences, National Autonomous University of Mexico. (Voucher number 186052). An ethanolic extract was made according to Ikken et al. [16], with some modifications. One hundred and fifty grams of leaves was washed with running water and left to dry for 24 h. Then, the leaves were macerated in a mortar with 1000 mL of absolute ethanol. The extraction was carried out at room temperature for 72 h, after which the material was vacuum filtered through a glass filter. The ethanol was evaporated by bubbling with nitrogen for 12 h, and the obtained soft, humid, sticky mixture was resuspended in 18 mL of DMSO and maintained at 4 °C until use. The ethanol extract was preferred because previous reports have revealed that polar volatile compounds have shown antimutagenic potential related to CYP inhibition [14,16,17,18]

### 2.3. Chemical Identification

The obtained extract was subjected to gas chromatography coupled with mass spectrometry (GC–MS) to identify its main components. For this purpose, solid-phase microextraction was applied to concentrate the volatile compounds, which were then injected into a Perkin-Elmer Clarus SQ8S instrument (Shelton, CT, USA) with a capillary column (HP-5MS) of size 30 m × 0.25 mm × 0.25 mm (J&W Agilent, Santa Clara, CA, USA). For the first three min, the temperature of the equipment was 40 °C, and the temperature was increased to 20 °C per minute to reach 300 °C for 10 min. Helium was used as a carrier at a flow rate of 1 mL/min, and the compounds were identified according to the Library of the National Institute of Standards and Technology.

### 2.4. The Ames Test

The study was carried out according to the indications of Maron and Ames [12], as well as with the recommendations expressed by the OCDE with respect to this assay [15]. The *Salmonella typhimurium* strains used were TA98, TA100, TA102, TA1535, and TA1537.

The reaction mixture included 100 µL of *Salmonella* culture (10^9^ bacteria) plus 10 µL of Piper auritum leaf ethanol extract (0.002739, 0.02739, 0.2739, 2.739, and 27.39 mg per plate) placed on top agar with traces of biotin and histidine. Another experiment used the same chemicals but used safrole (SA) instead of the extract. In this case, the concentrations used were 10, 50, 100, 500, and 1000 µg/plate. Both studies were performed without and with the inclusion of the S9 fraction. In the last case, the S9 fraction was mixed with 5 mM glucose-6 phosphate, 4 mM NADPH, 8 mM MgCl_2_, and phosphate buffer at pH 7.4. The reaction mixture with or without S9 was placed in Petri dishes containing minimum agar plus glucose and Vogel–Bonner salts and left at 37 °C for 48 h. Finally, the number of revertant colonies (His^+^) in each treatment was counted. Importantly, we used different mutagens as positive controls depending on the strain. For TA 98 and TA100, BaP with S9 and 2-NF without S9 was used; for TA1535, TA1537 and TA102 2-AA were used with S9; and sodium azide, picrolonic acid and 4-NQO were used without S9.

The antimutagenic assay was performed as previously described; however, in this case, we only used strain TA98, and the test compound in the *Salmonella* culture was BaP (10 µg/plate) plus the S9 fraction.

The extract and safrole were considered mutagenic when the number of induced revertant colonies doubled compared with that found in the control plates of each strain, and a dose-dependent effect was observed. The criterion for considering the extract and safrole as antimutagenic was a dose-dependent decrease in the number of revertant colonies found on the plates containing BaP with S9. The toxicity of the tested agents was assessed by observing background bacterial growth on minimal agar plates due to traces of histidine in the medium [19].

### 2.5. Bacterial Membranes Containing Human CYP1A1

Luria–Bertani/ampicillin (200 µg/mL) was used as the culture medium for *Escherichia coli* DH5α cells expressing human CYP1A1 and NADPH P450 reductase (kindly donated by Dr. Peter Guengerich, Vanderbilt University, Nashville, TN, USA). The expression of human CYP1A1 was achieved following the method of Parikh et al. [20] whereby cell cultures were diluted 1:100 in Terrific Broth/ampicillin medium (100 μg/mL) supplemented with 1 mM isopropyl β-D thiogalactoside, 0.5 mM aminolevulinic acid, 1 mM thiamine and trace salts. We followed the method proposed by Guo et al. [21] to obtain bacterial membranes that were preserved in potassium phosphate buffer (0.067 M, pH 7.4) supplemented with 6 mM magnesium acetate, 20% *v*/*v* glycerol, 10 mM 2-mercaptoethanol, and a 1× protease inhibitor cocktail at −80 °C until use. The protein content was estimated via the Bradford assay.

### 2.6. Kinetics of Human CYP1A1 Inhibition

The activity of human CYP1A1 was calculated via the method proposed by Burke et al. [22] with different concentrations of 7-ethoxyresorufin (0.15–5 μM) as the substrate in the absence or presence of safrole (0.10–0.75 µM). To determine the type of inhibition, an incubation volume of 200 μL containing human CYP1A1 bacterial membranes (80 μg) and 0.5 mM NADPH in buffer pH 7.6 (50 mM Tris–HCl and 25 mM MgCl_2_) was used.

### 2.7. Molecular Docking Assays

The 3D structure of human CYP1A1 (PDB ID: 4I8V, [23]) cocrystalized with the inhibitor α-naphthoflavone was retrieved from the Protein Data Bank [24] and prepared via the PyMOL Molecular Graphics System (version 2.5.7, Schrödinger, LLC), (New York, NY, USA) which removes counterions, crystallographic water, and other ligands (except for the heme group). MGLTools version 1.5.7 [25] was used to add atomic charges and solvation parameters. Docking simulations centered on the catalytic site of human CYP1A1 were conducted via AutoDock Vina version 1.2.0 [26] with safrole as the ligand (ChemSpider ID21106259, Royal Society of Chemistry). Binding conformation analysis was performed with PyMOL and MAESTRO (version 13.7, Schrödinger, LLC).

## 3. Results

### 3.1. Chemical Identification

The main phytochemicals present in the extract were identified via the GC–MS assay and are shown in Table 1. Fifteen compounds were identified, 93% of which were terpenes and a phenylpropanoid. Safrole was the most abundant chemical, reaching 68.7%. Other abundant compounds included γ-terpinene, 2-carene, copaene, and caryophyllene, which represented 20% of the identified components. The same table also shows the concentrations of some of the chemicals in the extract and confirms the important presence of safrole (364.53 ppm).

### 3.2. The Ames Test

The next step was to test the mutagenic potential of the plant extract and its main component safrole; we determined that none of the five bacterial strains used presented a duplication of spontaneous reversion or exhibited a dose–response relationship; the Salmonella/microsome mutagenicity assay results indicated that the ethanol extract had no mutagenic potential (Table 2). This conclusion is congruent with the expected and observed spontaneous reversion in the strains that were used, with the absence of toxicity determined for the plant extract and safrole, as well as with the number of revertants found for each of the specific positive controls. Table 3 shows the mutagenic potential of safrole. In this case, a similar response to what was described previously for the extract was observed. The testing of five concentrations (from 10 to 1000 µg/plate) revealed values in the range determined for the spontaneous mutation level of the strains used, indicating the absence of a mutagenic effect. In contrast, the six selected positive mutagens significantly increased the number of revertants, as expected.

**Figure 1:**
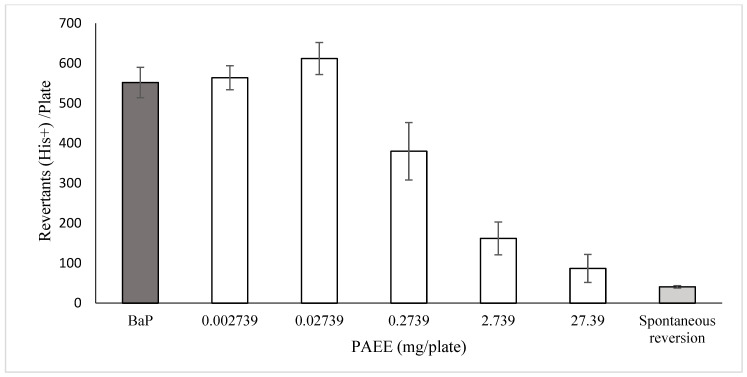
Antimutagenic potential of *Piper auritum* ethanol extract (PAEE) on the effect of BaP. *Salmonella*/microsome mutagenicity assay (TA98 strain with metabolic activation). Each bar represents the mean ± SDM of three individual plates. In the white bars, 1 mg/plate of PAEE was applied immediately after the incorporation of BaP (10 µg/plate). Spontaneous reversion denotes the mutation rate observed in the absence of mutagen.

**Figure 2:**
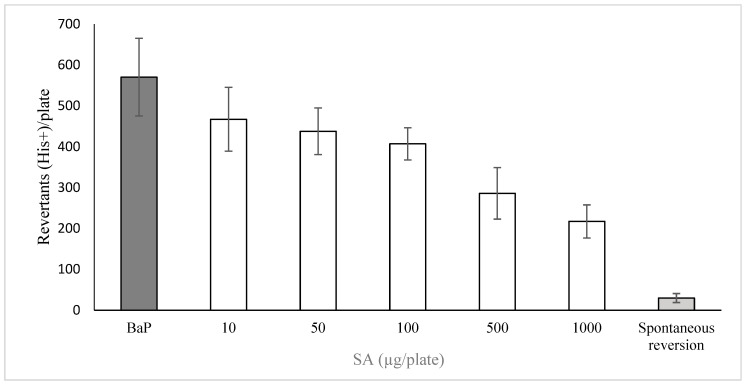
Antimutagenic potential of SA on the effect of BaP. *Salmonella*/microsome mutagenicity assay (TA98 strain with metabolic activation). Each bar represents the mean ± SDM of three individual plaques. In the white bars, 1 µg/plate of SA was applied immediately after the incorporation of BaP (10 µg/plate). Spontaneous reversion denotes the mutation rate observed in the absence of mutagen.

Our results concerning the antimutagenic capacity of the plant extract against the damage induced by BaP are presented in Figure 1. Here, we show a strong effect of the mutagen in comparison with what is observed with the spontaneous revertants; furthermore, no protection is obtained by treatment with the two low concentrations of the extract (0.002739 and 0.02739 mg/plate). However, the other three concentrations (0.2739, 2.739 and 27.39 mg/plate) significantly decreased the number of revertant colonies, from 37% to 90%. The colony reduction from the second to the last concentration corresponded to 179 revertant colonies/mg of extract. With respect to safrole, we also detected a concentration-dependent decrease in the number of colonies compared with the number induced by BaP (Figure 2). Our results demonstrated that the inhibitory capacity was observed with the first tested concentration and reached 65% at 1000 µg/plate (relative to the effect of BaP). Spontaneous revertants were similarly as low as those following treatment with the plant extract.

### 3.3. Kinetics of Human CYP1A1 Inhibition

**Figure 3:**
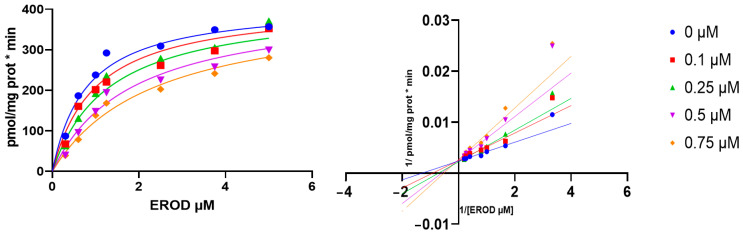
Inhibition kinetics of human CYP1A1 activity by safrole. Plots of velocity versus substrate concentration (**left panel**) and Lineweaver–Burk plots (**right panel**).

The previous result prompted us to explore the capacity of safrole to inhibit CYP activity. Figure 3 shows that safrole is able to inhibit EROD activity; nonlinear regression analysis and Lineweaver–Burk plots indicated competitive inhibition of safrole, with Ki, Km, and Vmax values of 0.38, 0.79, and 414.4 µM, respectively.

### 3.4. Molecular Docking Assays 

**Figure 4:**
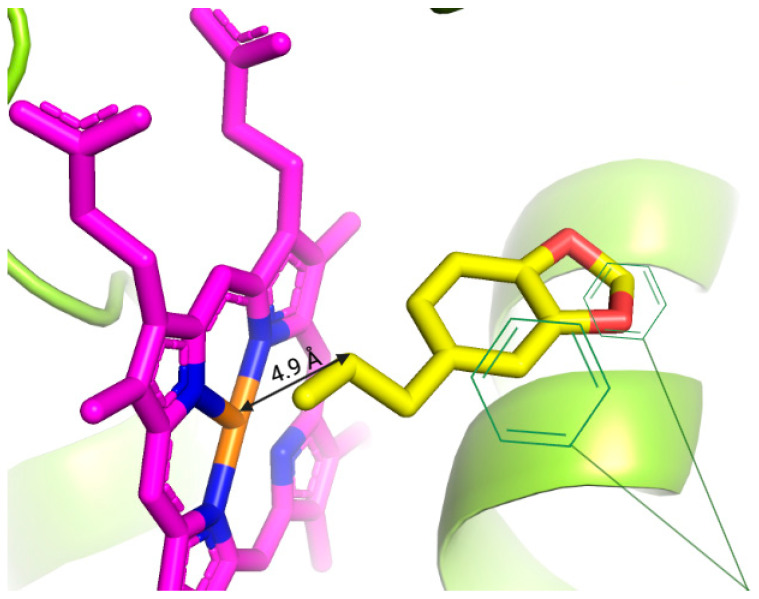
Molecular docking poses of safrole interacting with human CYP1A1. Protein–ligand interactions in the active site.

To explore a possible mechanism to explain the safrole antimutagenicity, we performed a docking analysis that revealed the preferred binding sites of safrole to the human CYP1A1 structure (Figure 4). After visual inspection of the top-ranked positions, a binding site with the heme group located in the active cavity of CYP1A1 was identified as the main site of interaction for safrole. Other important amino acids, such as Ser-122, Phe-123, Phe-224, Leu-312, Asp-313, Gly-316, Ala-317, Thr-321, Ile-386, Leu-496, Thr-497, and Hem-601, participate in all protein–ligand interactions with CYP1A1 (Table 4).

## 4. Discussion

The induction of gene mutation within a DNA sequence can result in an adverse impact, which may alter or inhibit gene function. In efforts to determine such alterations, the Ames test has been successfully applied; it is a reverse mutation assay that employs various *S. typhimurium* histidine-dependent strains with different types of mutations in the histidine operon. These mutations are briefly described in Table 5. Moreover, the test includes the incorporation of mammalian liver microsomes to enable the biotransformation of indirect mutagens [27].

In the case of plants or plant extracts with potential biological properties, the Ames test has become one of the most commonly used tests to determine mutagenic and antimutagenic effects. In this area of study, a systematic review of plants with biomedical capabilities revealed 478 species (mainly from the Asteraceae, Fabaceae, and Lamiaceae families), which included 18% with mutagenic effects, 82% with nonmutagenic effects, and 21% with antimutagenic potential [28].

**Table 5:** Characteristics of *Salmonella typhimurium* strains used in the Ames test.

Strain	Histidine Mutation	Reversion Event	LPS	DNA Excision Repair	Plasmid
TA98	D3052	Frameshift	*rfa*	*Δ uvrB*	pKM101
TA100	G46	Base-pair substitution	*rfa*	*Δ uvrB*	pKM101
TA1535	G46	Base-pair substitution	*rfa*	*Δ uvrB*	-
TA1537	C3076	Frameshift	*rfa*	*Δ uvrB*	-
TA102	G428	Transitions/transversions	*rfa*	Wild-type genes	pKM101, pAQ1

LPS: Partial lipopolysaccharide deletion that induces membrane permeability. (*Δ*) deletion of *uvrB* genes that block DNA excision repair. Plasmid: confers antibiotic resistance and increases sensitivity for mutagen detection. Adapted from Vijay et al. [29].

With respect to the *Piper* genus, the most widely studied species are *P. nigrum* and *P. betle*, which are among the approximately 106 species with biomedical properties. However, few species have been evaluated with the Ames test to determine mutagenic or antimutagenic potential, although a number of extracts from different parts of the plant are used worldwide in traditional medicine against a number of diseases, and reports have demonstrated that they contain secondary metabolites with deleterious or beneficial activities [30].

Our laboratory previously demonstrated the non-mutagenic potential of *P. auritum* and, in contrast, a strong antimutagenic effect of an ethanolic extract against food-borne heterocyclic amines, which we showed using the TA98 strain in the Ames test [14]. The absence of mutagenicity observed in the present report seems overwhelming because our previous result was confirmed in five different *S. typhimurium* strains, which included the detection of frameshift mutations and base pair substitutions. In addition, our results comply with the report by Vizoso et al. [30], who found a non-genotoxic effect of an ethanol extract of the plant in the Ames test, the somatic segregation test in *Aspergillus nidulans*, and in the mouse bone marrow micronucleus assay. Confirmation of the innocuous genotoxic role of *P. auritum* would be relevant for the safe use of the plant for nutritional and medicinal purposes.

Additionally, with the use of the TA98 *S. typhimurium* strain, we confirmed the strong antimutagenic potential of the tested extract against the damage induced by BaP, an effect that started with 0.2739 mg/plate and showed a concentration-dependent decrease of up to 90% with 27.39 mg/plate. With respect to the results obtained with the tested extract, all of the main compounds determined via the GS–MS assay have been reported to possess antioxidant, cytoprotective or beneficial health potential by themselves or as part of plant extracts or essential oils [31,32,33,34,35,36,37,38,39,40,41], suggesting that various compounds may participate in synergism to produce the observed effect. In this field, specific chemicals from plants have been reported with antimutagenic and antiproliferative properties, for example, punicalagin and ellagic acid from *Punica granatum* [42]. Considering the beneficial biological effects reported for other constituents present in the *P. auritum* extract, it is advisable to examine its specific antimutagenic potential and mechanism of action.

BaP is the main representative of polycyclic aromatic hydrocarbons and has been identified as a potent carcinogen. It is found in the air, surface water, cigarette smoke, and grilled and smoked food products, and it can be exhibited through occupational exposure. The compound is readily absorbed after inhalation, oral administration and through the skin; therefore, efforts to block its effects are of significance [43]. The antimutagenic properties of the plant extract and safrole reported here are congruent with this line of research, although studies using mammalian and in vivo assays should be conducted to confirm the observed effect. The BaP blocking biotransformation by safrole suggests that this effect may also occur with a number of carcinogens that require the same type of metabolic activation, and therefore, this field is open to diverse research approaches. In addition, it is known that CYP1A1 and CYP1A2 can metabolize a broad range of foreign compounds and have significantly overlapping substrate specificities, a behavior that suggests the pertinence of exploring molecular similarities and dissimilarities of safrole’s interaction with the enzymes [44].

Moreover, safrole is of specific interest because the compound has been identified as an alkenylbenzene that may be carcinogenic due to its ability to biotransform into reactive compounds [45,46]. Although no gene mutations were observed in rat hepatocytes treated with the chemical, Daimon et al. [47] detected the induction of DNA adducts and chromosomal aberrations. However, reports have also shown that the chemical has antioxidant, cytoprotective and beneficial health properties [10], findings that suggest complex behavior of safrole, which is likely related to the species that were studied and the experimental conditions used. In our present report, we determined a concentration-dependent antimutagenic effect of the compound on the damage induced by BaP, an indirect mutagen requiring biotransformation by the enzyme CYP1A1 to give rise to the deleterious compound benzo[a]pyrene-trans-7,8-dihydrodiol-9,10-epoxide. Safrole is also an indirect xenobiotic that can undergo complex biotransformation. Various enzymes of phase I in the biotransformation process may participate, mainly CYP1A2 but also CYP2A6, 2E1, 2C9, and 2D6, and they generate electrophilic metabolites such as safrole 2,3 epoxide, o-quinone and p-quinone methide that may form DNA adducts in addition to hydrosafrole, which, through sulfonation in phase II, generates 1-sulfosafrole that may give rise to covalent bonds with biomolecules and damage DNA [10]. Our docking studies established that safrole is able to interact with 11 types of amino acids and with the heme group of the active site of CYP1A1 by π−π stacking, which may sustain the observed overlapping substrate specificity among CYP isoforms, particularly 1A1 and 1A2, which are closely related [44,48]. The binding of safrole to CYP1A1 may be the antimutagenic mechanism because it results in a reduction in the biotransformation of BaP to the active epoxide. Our docking results are in accordance with those of Pedroni et al. [49], who reported that the related alkenylbencene apiole exhibited positive and relatively high docking scores when GOLD software (version 2021) was used for protein–ligand interactions. These findings suggest the potential of alkenylbencenes to interact favorably with the CYP1A1 active site.

Our findings are relevant in light of the importance of the CYP1A enzyme, which is involved in the bioactivation of a variety of carcinogens that can trigger the formation of DNA adducts, subsequent mutagenesis and tumor formation; CYP1A also has a role in the biotransformation of endogenous substances closely related to the occurrence and development of various human diseases and in the metabolic clearance of various anticancer drugs, making it critical for anticancer drug resistance [50].

## 5. Conclusions

In conclusion, by using the Ames/*Salmonella typhimurium* test, we showed here the lack of a mutagenic ability of the ethanolic extract of *P. auritum* and its main constituent, safrole; moreover, we confirmed that both of them exhibited strong antimutagenic ability against BaP, an ubiquitous polycyclic aromatic hydrocarbon that has been recognized by the International Agency for Research on Cancer as carcinogenic to humans (Group 1) [51]. Enzyme inhibition experiments revealed that safrole and the plant extract are possible CYP1A1 inhibitors, which was confirmed by docking analysis, confirming that safrole is a competitive inhibitor. Our present results may support new efforts to confirm mutagenic/antimutagenic findings with other tests, including mammalian and in vivo assays. Such studies may provide information for a secure understanding of the biological effects of plants. Evaluation of other plant constituents may also be undertaken to better understand their mutagenic/antimutagenic properties. Safrole is of particular importance in light of its classification as a carcinogen; therefore, it is mandatory to determine its mutagenic and genotoxic potential, as well as its DNA-damaging preventive properties through appropriate assays, including those aimed at confirming its mechanism of action.

## Figures and Tables

**Table 1:** Chemical identification and concentration of *Piper auritum* ethanol extract by the GC-MS assay.

Compounds	Retention Time (s)	Abundance (%)	Concentration (ppm)
Alfa-Pinene	5.473	1.25	-
Beta-Pinene	5.953	2.50	-
Carene	6.365	1.92	-
Gama-Terpinene	6.771	7.14	33.44
2-Carene	7.033	4.90	-
Safrole	8.654	68.71	364.53
Copaene	9.246	4.11	14.87
Gama-Muurolene	9.336	0.57	-
Cariophyllene	9.554	4.41	19.93
Humulene	9.764	1.0	-
Pentadecane	9.914	0.01	-
Germacrene	9.944	1.65	-
Muurolene isomere	10.019	0.29	-
Alfa-Copaene	10.041	0.62	-
B-Bisabolone	10.364	0.50	-

Each value represents the relative abundance identified by GC-MS.

**Table 2:** Absence of gene mutation by *Piper auritum* ethanol extract (PAEE). *Salmonella*/microsome mutagenicity assay.

mg/Plate	*S. typhimurium* (His^+^ Revertants)									
	TA98		TA100		TA1535		TA1537		TA102	
PAEE	(−S9)	(+S9)	(−S9)	(+S9)	(−S9)	(+S9)	(−S9)	(+S9)	(−S9)	(+S9)
0.002739	25 ± 2	36 ± 4	125 ± 17	124 ± 17	14 ± 2	15 ± 1	10 ± 3	11 ± 2	537 ± 14	567 ± 11
0.02739	28 ± 2	42 ± 6	122 ± 6	141 ± 11	17 ± 5	15 ± 2	10 ± 2	10 ± 2	562 ± 15	533 ± 27
0.2739	20 ± 1	41 ± 7	119 ± 7	133 ± 13	15 ± 6	17 ± 1	10 ± 3	10 ± 3	533 ± 15	540 ± 10
2.739	23 ± 4	40 ± 9	115 ± 13	131 ± 4	20 ± 9	16 ± 2	11 ± 3	12 ± 5	528 ± 5	548 ± 26
27.39	29 ± 5	33 ± 5	133 ± 11	122 ± 14	16 ± 5	14 ± 3	11 ± 4	10 ± 3	501 ± 24	557 ± 22
Spontaneous reversion	25 ± 3	41 ± 3	120 ± 13	119 ± 4	14 ± 3	11 ± 4	11 ± 3	9 ± 3	519 ± 6	572 ± 13
Positive mutagens	2419 ± 169 ^1^	765 ± 109 ^2^	1909 ± 428 ^1^	2399 ± 293 ^2^	481 ± 28 ^3^	371 ± 20 ^4^	63 ± 2 ^5^	120 ± 12 ^4^	2341 ± 149 ^6^	2080 ± 27 ^7^

Results represent the mean ± SDM of revertants observed in three individual plaques after a 48 h incubation period at 37 °C. The positive mutagens used were as follows: ^1^ 2-nitrofluorene (5 µg), ^2^ benzo(a)pyrene (10 µg), ^3^ sodium azide (1 µg), ^4^ aminoanthracene (2 µg), ^5^ picrolonic acid (100 µg), ^6^ 4-NQO (1 µg), ^7^ aminoanthracene (5 µg).

**Table 3:** Absence of gene mutation by safrole (SA). *Salmonella*/microsome mutagenicity assay.

μg/Plate	*S. typhimurium* (His^+^ Revertants)									
	TA98		TA100		TA1535		TA1537		TA102	
SA	(−S9)	(+S9)	(−S9)	(+S9)	(−S9)	(+S9)	(−S9)	(+S9)	(−S9)	(+S9)
10	19 ± 3	20 ± 2	110 ± 16	97 ± 18	16 ± 3	13 ± 1	10 ± 3	12 ± 3	502 ± 4	573 ± 6
50	22 ± 7	34 ± 8	120 ± 17	108 ± 18	16 ± 6	13 ± 4	14 ± 4	10 ± 3	489 ± 19	558 ± 14
100	27 ± 4	38 ± 9	107 ± 10	105 ± 9	20 ± 4	11 ± 1	10 ± 1	10 ± 1	484 ± 13	520 ± 10
500	18 ± 3	26 ± 9	100 ± 17	108 ± 9	19 ± 3	15 ± 5	12 ± 3	11 ± 4	451 ± 18	477 ± 17
1000	22 ± 9	36 ± 7	112 ± 6	102 ± 9	17 ± 2	18 ± 2	11 ± 4	9 ± 3	512 ± 40	504 ± 12
Spontaneous reversion	26 ± 5	29 ± 6	114 ± 9	103 ± 7	14 ± 3	11 ± 4	11 ± 3	9 ± 3	519 ± 6	572 ± 13
Positive mutagens	2070 ± 44 ^1^	476 ± 24 ^2^	1909 ± 428 ^1^	2399 ± 293 ^2^	481 ± 28 ^3^	371 ± 20 ^4^	63 ± 2 ^5^	120 ± 12 ^4^	2341 ± 149 ^6^	2080 ± 27 ^7^

Results represent the mean ± SDM of revertants observed in three individual plates after a 48 h incubation period at 37 °C. The positive mutagens used were as follows: ^1^ 2-nitrofluorene (5 µg), ^2^ benzo(a)pyrene (10 µg), ^3^ sodium azide (1 µg), ^4^ aminoanthracene (2 µg), ^5^ picrolonic acid (100 µg), ^6^ 4-NQO (1 µg), ^7^ aminoanthracene (5 µg).

**Table 4:** Interactions and blinding energy of ligands with human CYP1A1.

Compound	Structure	Enzyme (CYP1A1)		
		Binding Energy (kcal/mol)	Interactions	Distance to the Hem Group
Safrole	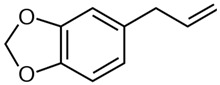	−7.879	Ser-122, Phe-123, Phe-224, Leu-312, Asp-313, Gly-316, Ala-317, Thr-321, Ile-386, Leu-496, Thr-497, Hem-601	4.9 Å

## Data Availability

Data will be made available on request.
